# A Minimally Invasive Technique for Short Spiral Implant Insertion with Contextual Crestal Sinus Lifting in the Atrophic Maxilla: A Preliminary Report

**DOI:** 10.3390/healthcare9010011

**Published:** 2020-12-24

**Authors:** Massimo Corsalini, Silvia D’Agostino, Gianfranco Favia, Marco Dolci, Angela Tempesta, Daniela Di Venere, Luisa Limongelli, Saverio Capodiferro

**Affiliations:** 1 Department of Interdisciplinary Medicine, University of Bari Aldo Moro, 70023 Bari, Italy; massimo.corsalini@uniba.it (M.C.); gianfranco.favia@uniba.it (G.F.); angelatempesta1989@gmail.com (A.T.); daniela.divenere@uniba.it (D.D.V.); luisannalimongelli@gmail.com (L.L.); 2 Department of Medical, Oral and Biotechnological Sciences, University of Chieti-Pescara, 66100 Chieti, Italy; silviadagostino00@gmail.com (S.D.); marco.dolci@unich.it (M.D.)

**Keywords:** short dental implant, spiral implant, atrophic posterior maxilla, maxillary sinus, maxillary crestal sinus lifting

## Abstract

The most recently reported techniques for the rehabilitation of the atrophic posterior maxilla are increasingly less invasive, as they are generally oriented to avoid sinus floor elevation with lateral access. The authors describe a mini-invasive surgical technique for short spiral implant insertion for the prosthetic rehabilitation of the atrophic posterior maxilla, which could be considered a combination of several previously described techniques based on the under-preparation of the implant site to improve fixture primary stability and crestal approach to the sinus floor elevation without heterologous bone graft. Eighty short spiral implants were inserted in the molar area of the maxilla in patients with 4.5–6 mm of alveolar bone, measured on pre-operative computed tomography. The surgical technique involved careful drilling for the preparation of implant sites at differentiated depths, allowing bone dislocation in the apical direction, traumatic crestal sinus membrane elevation, and insertion of an implant (with spiral morphology) longer than pre-operative measurements. Prostheses were all single crowns. In all cases, a spiral implant 2–4 mm longer than the residual bone was placed. Only two implants were lost due to peri-implantitis but subsequently replaced and followed-up. Bone loss values around the implants after three months (at the re-opening) ranged from 0 to 0.6 mm, (median value: 0.1 mm), while after two years, the same values ranged from 0.4 to 1.3 mm (median value: 0.7 mm). Clinical post-operative complications did not occur. After ten years, no implant has been lost. Overall, the described protocol seems to show good results in terms of predictability and patient compliance.

## 1. Introduction

Growing evidence exists in the literature regarding the use of short dental implants (usually considered as <10 mm in length) for the prosthetic rehabilitation of jaws with reduced vertical bone dimension [1,2,3,4,5,6,7,8]. Generally, one of the most challenge clinical condition for implant placement is surely the atrophic posterior maxilla, usually after teeth extraction or jaw surgery, and when the residual bone measures 4.5–6 mm in height on radiograms [1,3,8]. Over time, many surgical techniques as well implant morphologies have been suggested, mostly aimed to avoid sinus floor elevation in such situations, as the latter is frequently characterized by high postoperative morbidity and costs, as well as delayed prosthetic rehabilitation [1,2,3]. Surely, intraoperative (mostly tearing of the Schneiderian membrane as occurring in 7–44% of the procedures, in addition to antral or nasal penetration, bleeding, fenestration, dehiscence or perforation of the alveolar bone, insufficient implant primary stability, etc.) and postoperative complications (pain, swelling, bleeding, edema, hemosinus, sinus infection, sinusitis, graft or fixture migration or loss, oroantral fistula, etc.) are the main factors to consider when a maxillary sinus augmentation is required for implant placement [4,5,6].

In the last two decades, the use of minimally invasive techniques is becoming increasingly widespread in all fields of dentistry, and growing evidence in the literature supports such use [7,8].

The current study describes a minimally invasive surgical procedure for the insertion of 80 short spiral implants in the posterior atrophic maxilla (all for single tooth restorations) using a surgical technique of progressive steps that promotes crestal sinus lifting, with a follow-up of 10 years.

## 2. Materials and Methods

The main inclusion criteria were a residual vertical bone height minimum of 4.5 mm (with a range of from 4.5 to 6 mm) as measured on radiograms (computed tomography (CT) or cone beam computed tomography (CBCT)). No data regarding osteoporosis and possible medical treatments in females were collected, as such information was deemed unnecessary. There were 49 males and 31 females patients with an age range of 27–63 y.o. (median of 37 y.o.). Patients taking oral or i.v. bisphosphonates were excluded from the study. Surgery was conventional (scalpel incision, periosteal flap elevation and stitches) in 30 cases and flapless in the remaining 50 (with software assisted surgical guides—Modelguide, Bionova s.r.l., Italy), always with low-speed drilling and irrigation; adjunctive bio-material has never been used as bone graft. Surgical and prosthetic procedures were performed by the same oral surgeon (SC) to avoid interpersonal differences.

The surgical technique included the following steps:

- Step one—the first drill (2 mm in diameter) was used until the sinus floor was reached with atraumatic perforation, the latter probed with a gauge, and the negative status of Valsalva’s test was continuously checked;
- Step two—the second drill (2.8 mm in diameter) was used only for a depth of 2–4 mm;
- Step three—the third drill (3.2 mm in diameter) was used for only 2 mm in depth;
- Step four (optional, only for insertion of an implant of 5 mm in diameter)—the fourth drill (4.1 mm in diameter) was used for 1 mm in depth.

As for the follow-up, peri-implant bone loss was measured directly on a periapical radiograph (as peri-implant bone loss from the fixture–abutment connection to the depth) at the time of re-opening (second surgery for submucosal implants) and two years after prosthetic rehabilitation. Data regarding implant survival after ten years were mostly collected by phone recall (asking the patient if implant rehabilitation was still present or not) as some patients refused additional radiograph or clinical follow-up; thus, data regarding bone loss measurement after ten years are missing.

## 3. Results

**Figure 1:**
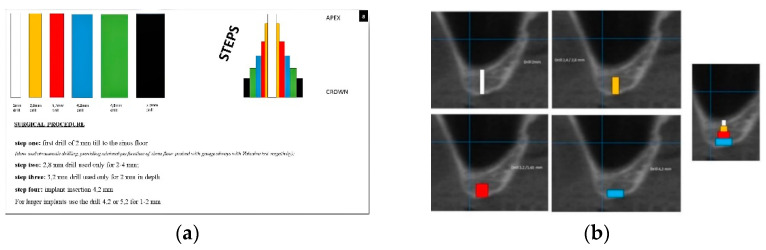
(**a**,**b**) Schematic description of the surgical technique allowing the formations of steps after drilling (**a**); as an example, the scheme is superimposed on a cone beam computed tomography (CBCT) of an atrophic posterior maxilla (**b**).

The surgical procedure is explained in detail in Figure 1a,b.

In all cases, a longer implant (from 2 to 4 mm) than the pre-operative radiological measures was positioned. Only two implants both placed by flapless surgery and with healing cap positioning were lost before the prosthetic rehabilitation, but they were repositioned after 45 days.

**Figure 2:**
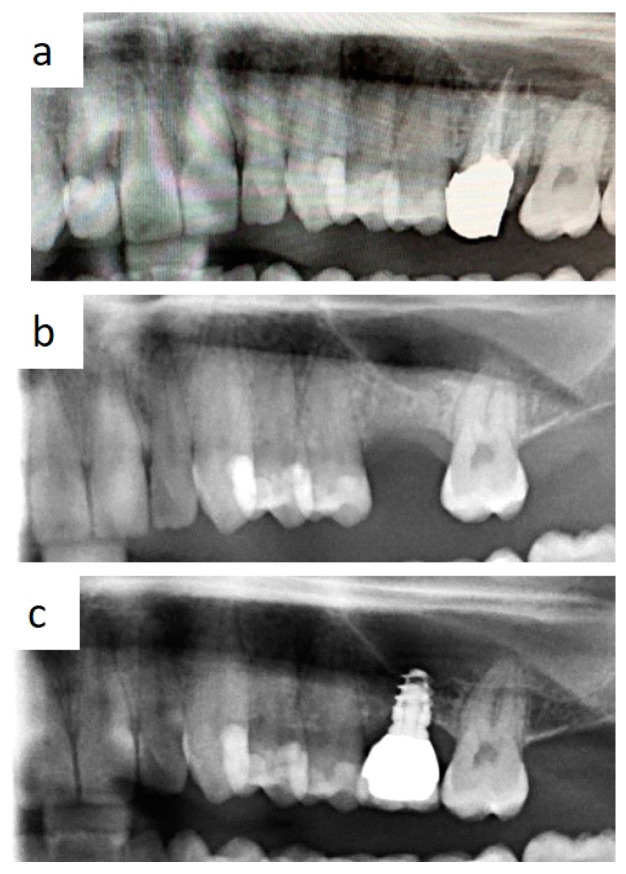
Occurrence of severe atrophy after removal of decayed first molar with periapical lesions (**a**,**b**); single tooth prosthetic rehabilitation without conventional sinus lift is feasible with short implant use (**c**).

**Figure 3:**
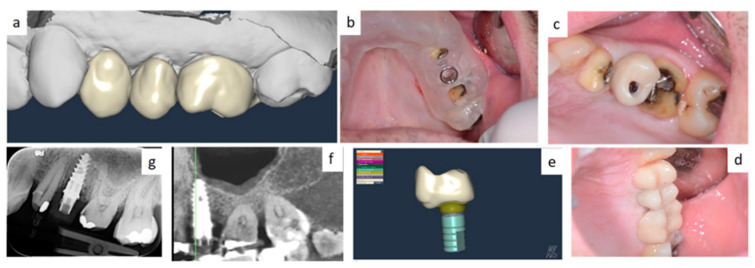
Single tooth restoration by screw-retained crown digitally planned by computer aided design (CAD) and CBCT (**a**); implant insertion was assisted by a surgical guide (**b**–**e**); fixture position was pre-operatively planned by simulation of crestal sinus membrane elevation (**f**,**g**).

**Figure 4:**
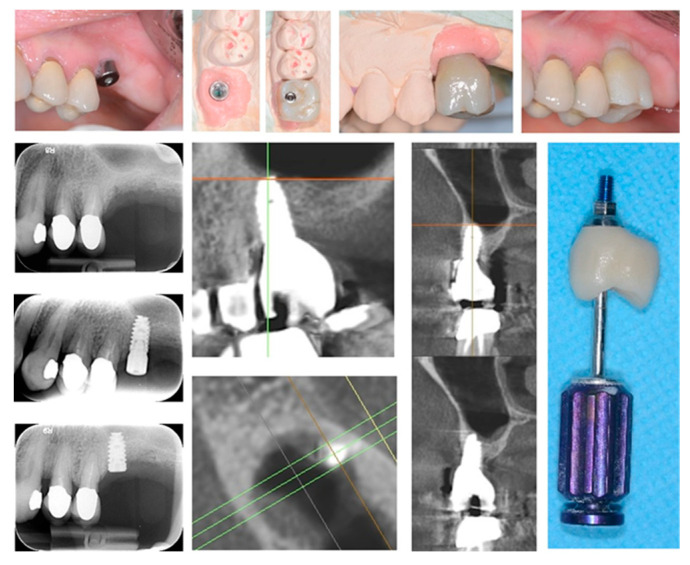
Implant-supported single tooth restoration in atrophic posterior maxilla with all of the pre- and post-surgical phases until final screw-retained prosthetic rehabilitation.

**Figure 5:**
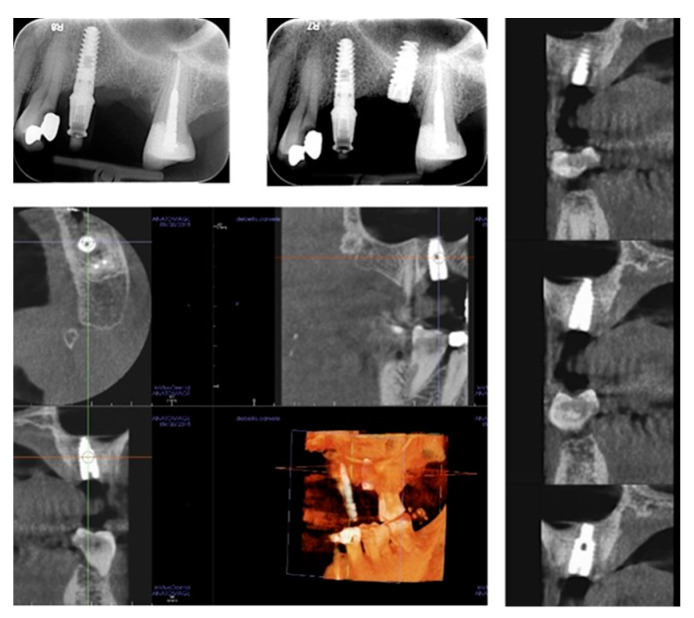
Radiological investigations showing the pre-surgical planning of the crestal sinus lifting and the radiological result after implant insertion; the elevation of sinus membrane is evident, and no signs of intra-sinusal inflammation are detectable after surgery.

In all cases, fixtures were placed with high insertion torque (up to 50 Ncm) and with atraumatic crestal lifting of the sinus membrane (Figure 2a–c and Figure 3a–f). Prostheses were placed 3 months after surgery; more precisely, 40 were cemented metal–ceramic crowns, and the remaining were screwed hybrid ceramic crowns (Vita Enamic Multicolor) with glued titanium bases (Figure 4). All implants had a diameter of 4.2 mm, and 35 were 6.25 mm in length, while the remaining 45 had a length of 8 mm (Figure 5).

Overall, a good peri-implant bone level was observable throughout the follow-up in all cases.

**Figure 6:**
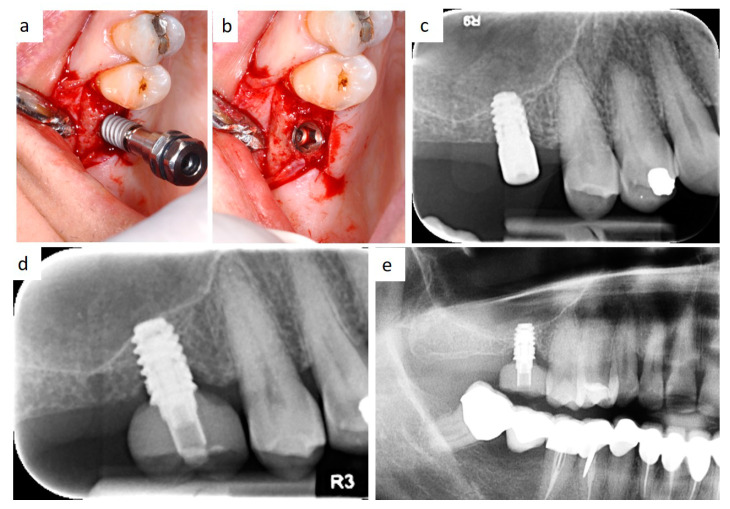
Intraoperative view of implant insertion by muco-periosteal flap elevation (**a**,**b**); the implant diameter is larger than implant site preparation, thus promoting bone dislocation in the apical direction by the self-tapping capability of the fixture and high primary stability. Radiological follow-up 3 months after insertion (**c**), 2 years after prosthetic rehabilitation (**d**), and after 10 years (**e**).

**Figure 7:**
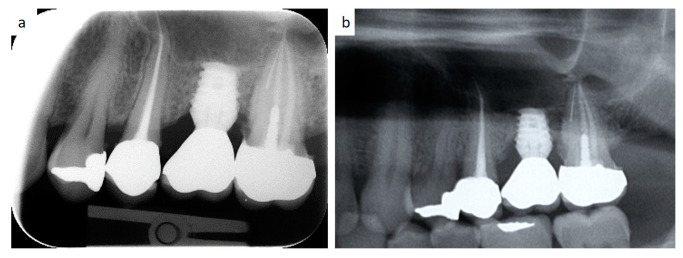
Periapical radiograms showing the bone loss at a 2-year (**a**) and a 10-year follow-up (**b**) around a short spiral implant (6.25 × 4.2 mm in diameter) supporting a single metal–ceramic crown.

Bone loss around implant was measured at the time of second surgery (three months later) with healing cup positioning (values ranging from 0 to 0.6 mm; median value: 0.1 mm) and also after two years (values ranging from 0.4 to 1.3 mm; median value: 0.7 mm). Clinical post-operative complications did not occur. (Figure 6a–e and Figure 7a,b) To date, no implant failure has occurred during the ten-year follow-up, as summarized in Table 1.

## 4. Discussion

Molar areas of the maxilla frequently show severe vertical loss of the alveolar bone after tooth removal. Vertical dimensions of 4–6 mm of residual bone are difficult to treat when implant insertion is required [1,2,5]. Various techniques have overtime been reported in the literature to allow implant insertion in such atrophic areas and especially to avoid sinus floor elevation by lateral access in addition to the related possible complications [6,7,8].

Moreover, the global time of rehabilitation (from the implant insertion to the final prosthetic rehabilitation) is certainly reduced, as it decreases from about 10–12 months for conventional sinus elevation to about 3–4 months without it [3,4,6].

Notably, in the last two decades there has been a general tendency to make such treatments less and less invasive thanks to both the innovation in implant design and superficial treatments of the fixtures [1,2,9].

Overall, the surgical technique described in this preliminary report is a combination of previously reported techniques of under-preparation of the implant sites aimed to improve fixture primary stability and crestal approach to sinus floor elevation as an alternative to the conventional lateral window, regardless of the thickness of the sinus membrane and always without heterologous bone graft [10,11,12,13,14,15,16]. More precisely, the technique we used is essentially based on a careful drilling at different depths (steps) of the residual alveolar bone in order to obtain a minimal implant site preparation, thus creating progressive steps. Subsequently, the high torque insertion of a self-tapering fixture with spiral morphology and with a smaller diameter than the preparation promotes bone displacement in the apical direction following traumatic sinus membrane elevation, thus allowing the possibility to insert a longer fixture (from 2 to 4 mm) than the measurements detected on radiographs.

In fact, it is generally accepted that spiral implants, due to their morphology (and also when of reduced length), promote bone dislocation during insertion towards the bottom of the implant site with an increase in the exploitable vertical dimension, contextually leading to both the aforementioned placement of a longer implant and to a high primary stability of the fixture [6,7,8,9,14,15,16,17,18].

In the past, some concerns regarding complications due to the increased crown–implant ratio in prosthetic rehabilitation supported by short implants were reported. In this regard, the recent literature demonstrated that no correlation exists between occurrence of biological and technical complications and the crown–implant ratio of implant-supported reconstructions, in addition to the fact that the crown–implant ratio does not influence peri-implant crestal bone loss [19,20,21,22]. Besides, several studies reported on the efficacy of short implants compared to 10 mm or longer implants placed in crestally lifted sinuses, also showing no significant differences regarding prosthesis and implant failures, complications, and radiographic peri-implant marginal bone lose [1,2,3,9,10,15,16,17,18,19].

Regarding the data literature, some recent studies (systemic reviews and meta-analyses) on short implants used for the rehabilitation of the atrophic maxilla were published by Ravidà A. et al. and Yan Q. et al. in 2019, [1,3]. In both studies, the authors agree on the comparable survival rate of maxillary short implants in comparison to longer implants, as well on the reduced biological complications, reduction of the rehabilitation time and costs and marginal bone loss. Nevertheless, the high variability of the fixture design, length and diameter, operator-related surgical technique, timing and materials of the prosthetic rehabilitation and possible parafunctional habits, in our opinion, are important variables which may highly influence data collection as well as comparisons.

Therefore, despite the limitations of the current study—especially those related to the low number of patients/implants and the missing comparable data/cases and follow-up values of bone loss for a statistical comparison—the therapeutic protocol herein described, including implants with spiral morphology and placement of progressive steps, seem to show good outcomes in terms of mini-invasiveness, predictability and acceptability by patients for single tooth restoration of the posterior atrophic maxilla. Further studies are needing to confirm or improve data regarding the long-term effectiveness and safety of short dental implants for the prosthetic rehabilitation of the atrophic maxilla.

## Figures and Tables

**Table 1:** Values of peri-implant bone loss during follow-up.

Implants	Peri-Implant Bone Loss at the Second Surgery	Peri-Implant Bone Loss Two Years Later	10-Year Follow-Up
35 fixtures 4.2 ∅ X 6.25 L mm	range: 0–0.6 mm (median: 0.1)	range: 0.4–1.3 mm (median: 0.7)	Yes
45 fixtures 4.2 ∅ X 8 L mm			Yes

∅: fixture diameter; L: fixture length.

## Data Availability

The data are not publicly available due to privacy reasons.

## References

[1] Yan Q., Wu X., Su M., Hua F., Shi B. (2019). Short implants (≤6 mm) versus longer implants with sinus floor elevation in atrophic posterior maxilla: A systematic review and meta-analysis. BMJ Open.

[2] Gürlek Ö., Kaval M.E., Buduneli N., Nizam N. (2019). Extra-short implants in the prosthetic rehabilitation of the posterior maxilla. Aust. Dent. J..

[3] Ravidà A., Wang I.C., Sammartino G., Barootchi S., Tattan M., Troiano G., Laino L., Marenzi G., Covani U., Wang H.L. (2019). Prosthetic Rehabilitation of the Posterior Atrophic Maxilla, Short (≤6 mm) or Long (≥10 mm) Dental Implants? A Systematic Review, Meta-analysis, and Trial Sequential Analysis: Naples Consensus Report Working Group A. Implant. Dent..

[4] Kim J., Jang H. (2019). A review of complications of maxillary sinus augmentation and available treatment methods. J. Korean Assoc. Oral Maxillofac. Surg..

[5] Schwartz-Arad D., Herzberg R., Dolev E. (2004). The Prevalence of Surgical Complications of the Sinus Graft Procedure and Their Impact on Implant Survival. J. Periodontol..

[6] Proussaefs P., Lozada J., Kim J., Rohrer M.D. (2004). Repair of the perforated sinus membrane with a resorbable collagen membrane: A human study. Int. J. Oral Maxillofac. Implant..

[7] Cassetta M., Altieri F., Di Giorgio R., Barbato E. (2018). Palatal orthodontic miniscrew insertion using a CAD-CAM surgical guide: Description of a technique. Int. J. Oral Maxillofac. Surg..

[8] Cassetta M., Altieri F., Pandolfi S., Giansanti M. (2017). The combined use of computer-guided, minimally invasive, flapless corticotomy and clear aligners as a novel approach to moderate crowding: A case report. Korean J. Orthod..

[9] Sierra-Sánchez J.L., García-Sala-Bonmatí F., Martínez-González A., García-Dalmau C., Mañes-Ferrer J.F., Brotons-Oliver A. (2016). Predictability of short implants (<10 mm) as a treatment option for the rehabilitation of atrophic maxillae. A systematic review. Med. Oral Patol. Oral Cir. Bucal..

[10] Herrero-Climent M., Lemos B.F., Herrero-Climent F., Falcao C., Oliveira H., Herrera M., Gil F.J., Ríos-Carrasco B., Ríos-Santos J.V. (2020). Influence of Implant Design and Under-Preparation of the Implant Site on Implant Primary Stability. An In Vitro Study. Int. J. Environ. Res. Public Health.

[11] Salgar N. (2020). Osseodensified Crestal Sinus Window Augmentation: An Alternative Procedure to the Lateral Window Technique. J. Oral Implant..

[12] Boyacıgil D.U., Er N., Karaca Ç., Koç O. (2020). The effect of residual bone height and membrane thickness on sinus membrane perforation in crestal sinus grafting: A prospective clinical study. Int. J. Oral Maxillofac. Surg..

[13] Soardi C.M., Soardi B., Wang H.-L. (2020). Crestal Window Sinus Lift and Its Long-Term Clinical Outcomes. Int. J. Periodontics Restor. Dent..

[14] Arab H.R., Moeintaghavi A., Shiezadeh F., Nezhad M.H. (2018). Crestal Sinus Floor Elevation with Autogenous Press-Fit Dowel Bone Harvested Using Trephine Drills: A New Method. J. Long Term Eff. Med. Implant..

[15] Lorenz J., Blume M., Korzinskas T., Ghanaati S., Sader R.A. (2019). Short implants in the posterior maxilla to avoid sinus augmentation procedure: 5-year results from a retrospective cohort study. Int. J. Implant. Dent..

[16] Zhang X.M., Shi J.Y., Gu Y.X., Qiao S.C., Mo J.J., Lai H.C. (2017). Clinical Investigation and Patient Satisfaction of Short Implants Versus Longer Implants with Osteotome Sinus Floor Elevation in Atrophic Posterior Maxillae: A Pilot Randomized Trial. Clin. Implant. Dent. Relat. Res..

[17] Nedir R., Nurdin N., Najm S.A., El Hage M., Bischof M. (2017). Short implants placed with or without grafting into atrophic sinuses: The 5-year results of a prospective randomized controlled study. Clin. Oral Implant. Res..

[18] Fan T., Li Y., Deng W.W., Wu T., Zhang W. (2017). Short Implants (5 to 8 mm) Versus Longer Implants (>8 mm) with Sinus Lifting in Atrophic Posterior Maxilla: A Meta-Analysis of RCTs. Clin. Implant. Dent. Relat. Res..

[19] Cenkoglu B.G., Balcioglu N.B., Ozdemir T., Mijiritsky E. (2019). The Effect of the Length and Distribution of Implants for Fixed Prosthetic Reconstructions in the Atrophic Posterior Maxilla: A Finite Element Analysis. Materials.

[20] Blanes R.J. (2009). To what extent does the crown-implant ratio affect the survival and complications of implant-supported reconstructions? A systematic review. Clin. Oral Implant. Res..

[21] Capodiferro S., Favia G., Scivetti M., De Frenza G., Grassi R. (2006). Clinical management and microscopic characterisation of fatique-induced failure of a dental implant. Case report. Head Face Med..

[22] Nunes M., Almeida R.F., Felino A.C., Malo P., de Araújo Nobre M. (2016). The Influence of Crown-to-Implant Ratio on Short Implant Marginal Bone Loss. Int. J. Oral Maxillofac. Implant..

